# Phase II trial on SBRT for unresectable liver metastases: long-term outcome and prognostic factors of survival after 5 years of follow-up

**DOI:** 10.1186/s13014-018-1185-9

**Published:** 2018-11-26

**Authors:** Marta Scorsetti, Tiziana Comito, Elena Clerici, Ciro Franzese, Angelo Tozzi, Cristina Iftode, Lucia Di Brina, Pierina Navarria, Pietro Mancosu, Giacomo Reggiori, Antonella Fogliata, Stefano Tomatis, Guido Torzilli, Luca Cozzi

**Affiliations:** 1 Radiotherapy and Radiosurgery Department, Humanitas Cancer Center, Humanitas Clinical and Research Center, Via Manzoni 56, 20089 Rozzano, Milan Italy; 2 Department of Biomedical Sciences, Humanitas University, Via Manzoni 113, 20089 Rozzano, Milan Italy; 3 Department of Hepato-biliary Surgery, Humanitas Clinical and Research Center, Via Manzoni 56, 20089 Rozzano, Milan Italy; 4 Radiotherapy and Radiosurgery Department, Humanitas Research Hospital and Cancer Center, Via Manzoni 56, 20089 Milan, Rozzano Italy

**Keywords:** Metastatic liver cancer, SBRT, SABR, Volumetric modulated arc therapy

## Abstract

**Background:**

The aim of this study was to evaluate long-term efficacy and survival prognostic factors of stereotactic body radiation therapy (SBRT) for un-resectable liver metastases in patients enrolled in a prospective phase II trial.

**Methods and materials:**

5-year local control (LC), overall survival (OS), progression free survival (PFS) and toxicity rates were analyzed in patients with un-resectable liver metastases enrolled in a Phase II Trial on liver SBRT, with a prescription dose of 75Gy in 3 consecutive fractions.

**Results:**

A total of 61 patients with 76 lesions were enrolled, with a median follow-up time of 6.1 years. One, three and 5 year LC rates were 94 ± 3.1%, 78.0 ± 5.9% and 78.0 ± 5.9%, without reaching the median LC time. Median OS was 27.6 months and the survival rates were 85.2 ± 4.5%, 31.1 ± 5.9% and 18.0 ± 4.9% at 1, 3 and 5-year after SBRT, respectively. Univariate analysis showed that favorable primary site (colorectal, breast and gynecological) of metastases (*p* = 0.001) improved survival. Toxicity was moderate. One patient experienced G3 late chest wall pain, which resolved within 1 year from SBRT. No cases of Radiation Induced Liver Disease (RILD) were detected.

**Conclusions:**

Long-term results of this Phase II study suggest the efficacy and safety of SBRT for un-resectable liver metastases after 5-year of follow up. Selection of cases with positive prognostic factors may improve long-term survival of these oligo-metastastic patients and may confirm the role of SBRT as an effective alternative local therapy for liver metastases.

## Background

Early diagnosis, surgical techniques, effective chemotherapy regimens and the introduction of new ablative local therapy have significantly increased quality of life and overall survival of patients with cancer [1]. Multidisciplinary management of cancer led to an increased accrual of patients with few organ-confined metastases, who can experience long-term survival [2].

Liver is one of the most common sites of oligometastatic disease from several cancers, such as colorectal, breast and lung cancer. When metastatic disease from colorectal cancer (CRC) is confined to the liver, surgical resection represents the standard of care and it is associated with a better prognosis [3–6]. Approximately 70–90% of liver metastases are however un-resectable and a safe and effective alternative therapeutic option is necessary.

Minimally invasive local approaches were introduced as an alternative to surgery, including radiofrequency ablation (RFA) and microwave ablation (MWA). Nevertheless, these techniques have limits, mostly related to lesion size and location (lesions greater than 3–4 cm in diameter or located in proximity of major blood vessels, biliary tract, gallbladder or just beneath the diaphragm) [7, 8].

An increasing number of prospective trials on liver stereotactic body radiation therapy (SBRT) were published, with encouraging results in terms of local control (LC), toxicity and overall survival (OS) [9–17]. Relevant factors affecting the efficacy of SBRT were identified and included tumor size, prescription dose and histology [12, 18–23].

The aim of this study was to analyze long-term results of liver SBRT and to confirm the safety and the efficacy of this ablative therapy in the treatment of these oligometastatic patients with a complete 5-year follow-up.

## Methods and materials

### Patient cohort

Phase 2 prospective trial on SBRT for un-resectable liver oligo-metastases was approved by the internal ethical committee of Humanitas Cancer Center Hospital in 2010. Inclusion criteria of this observational study were described in previous publications [16, 17] and summarized in Table 1.

**Table 1:** Inclusion criteria

Inclusion criteria	
• Liver metastases considered not suitable for surgery, because of being technically or medically inoperable or because of patient refusal; • Maximum tumor diameter less than 6 cm; • No more than 3 liver lesions; • Normal liver volume greater than 1000 cm3; • No evidence of progressive or untreated gross disease outside of the liver; • No prior radiation therapy to the targeted area; • Adequate liver function, defined as total bilirubin < 3 mg/dL, albumin > 2.5 g/dL, normal prothrombin time (PT)/partial thromboplastin time (PTT) unless on anticoagulants, and serum levels of aspartate aminotransferase (AST) and alanine amynotransferase (ALT) less than 3 times the upper limit of normal; • No concurrent chemotherapy allowed, either within 14 days before SBRT or until the first revaluation thereafter; • No active connective tissue disorders; • Karnofsky Performance Status 70; • Minimum age 18 years; • Written informed consent.	

### SBRT technique

SBRT technique and assessment of efficacy and morbidity were described in previous publications [16, 17]. Patients were immobilized with a thermoplastic body mask, including a Styrofoam block for abdominal compression. A contrast-free and triple-phases contrast-enhanced computed tomography (CT) scan were acquired for all patients. The 4-dimensional CT (4D-CT) imaging was performed in patients with respiratory excursion greater than 5 mm. In most of patients, planning CT images were co-registered with magnetic resonance imaging (MRI) or positron emission tomography (PET) to better identify the target volume. The clinical target volume (CTV) was defined as equal to the gross tumor volume (GTV). In all patients who underwent 4D-CT scan, an internal target volume (ITV) was defined as the envelope of all CTVs in the different respiratory phases. The planning target volume (PTV) was generated from either the CTV or the ITV by adding an isotropic margin of 5 mm from ITV or of 7-10 mm in the cranial-caudal axis and 4-6 mm in the anterior-posterior and lateral axes from CTV. The plan objective was to cover at least 98% of the CTV (ITV) volume with 98% of the prescribed dose (V_98%_ = 98%) and for the PTV to cover 95% of the volume with 95% of the dose (V_95%_ = 95%). Prescription dose was 75Gy in 3 consecutive daily fractions of 25Gy each, prescribed as the mean dose to PTV. An individualized dose scheme, where the prescription dose might be reduced in a step-wise manner, was employed to respect dose constraints for organs at risk (OARs). Simulation phase, treatment planning, dose prescription, dose constraints for OARs and treatment delivery were summarized in Table 2. No specific constraints were applied in terms of conformality index. Dose gradient was controlled by means of the normal tissue objective tool as suggested in the table. Treatment was delivered in free breathing on a Varian TrueBeam linear accelerator using a 10 MV Flattening Filter Free beam with a maximum nominal dose rate of 2400 MU/minute with the RapidArc technique. Two coplanar partial arcs were used for the plans as a class solution. Three dimensional image guided radiation therapy (IGRT) was performed before each daily session to check patient and tumor position by means of cone beam CT (CBCT). In most of patients we identified and matched the bone land marks and soft tissue matching from the CBCT data with respect to the planning CT. In some patients who had previous surgery, surgical clips were used as markers to enhance the quality of the task.

**Table 2:** Treatment planning and delivery characteristics

Patient position	
• Supine position with arms above the head	
Immobilization device	
• Thermoplastic body mask • Styrofoam block for abdominal compression	
CT scan	
• Contrast-free and 3 phases contrast-enhanced • Acquisition in free quiet breathing mode • 3-mm slice thickness	
4-dimensional CT (4DCT)	
• In case of lesions located in the postero-superior segments, or with a shift greater than 5 mm on the CT scan	
GTV delineation	
• CT scan • Co-registration with magnetic resonance imaging (MRI) or positron emission tomography (PET)	
ITV and PTV delineation	
• For patients with 4D-CT, an internal target volume (ITV) was defined as the envelope of all GTVs in the different respiratory phases. • PTV = ITV + overall isotropic margin of 5 mm • PTV = GTV + 7–10 mm in the cranial-caudal axis and 4–6 mm in the anterior-posterior and lateral axes	
Prescription dose	
• 75 Gy/ 3 fractions (Full dose) • 67.5 Gy/ 3 fractions (Reduction of 10%) • 60 Gy/ 3 fractions (Reduction of 20%) • 52.5 Gy/ 3 fractions (Reduction of 30%)	
Dose constraints for OARs	
• Healthy liver: V15 Gy (volume receiving 15 Gy) < (total liver volume - 700 cm3) • Spinal cord: D 0.1 cm3 < 18 Gy • Both kidneys: V15 Gy < 35% • Duodenum, small bowel, esophagus, and stomach: V21 Gy < 1% • Heart: V30 Gy < 1% • Ribs: D30 cm3 < 30 Gy • dose fall off: from 100 to 30% in 5 mm	
IGRT system	
• Megavoltage CBCT before each daily session	

### Trial end points and response assessment

Primary end-point was to assess in field LC. Secondary end points were to define radiation treatment-related toxicity and OS.

Tumor response was defined according to European Organization for Research and Treatment of Cancer Response Evaluation Criteria In Solid Tumors (EORTC-RECIST) 1.1 [24]. After SBRT, physical examination and basal blood chemistry analyses were performed 21 days after and then every 2 months. Radiological restaging was performed every 3 months for 3 years and every 6 months for the following 2 years, if patients were disease-free. Acute and late toxicity were scored by the Common Terminology Criteria for Adverse Events 3.0.

Considering local control as primary endpoint, the study was designed to exclude a local control < 60% with an α-error of 5% and to demonstrate a LC > 80% with a power of 90%. This required the enrollment of 44 patients, with at least 33 disease local control was necessary. Actuarial LC, OS and PFS curves were generated by using the Kaplan-Meier method. Log rank test was used for group comparison. Survival was measured from the end of SBRT. Univariate analysis was used to correlate morphologic and clinical factors to LC, OS and PFS and statistical significance was accepted for *p*-values of < 0.05. A multivariate analysis was not performed due to low number of events in this cohort of patients. All analyses were performed using STATA version 13 software.

## Results

Sixty-one patients with 76 liver metastases were treated with SBRT between February 2010 and September 2011 in this phase II study. Baseline patients and treatment characteristics are previously described in a published paper on preliminary results [16, 17]. Twenty-eight patients (45.9%) received liver-directed therapy before the SBRT treatment. Particularly, 21 patients were treated with hepatic surgery, 2 with RFA and 5 with both. Twenty-nine patients (47.5%) were treated for CRC liver metastases, 11 (18%) for breast cancer hepatic lesions and 7 (11.5%) for gynecological cancer liver metastases. Other primary tumors were exocrine pancreas in 5 patients, lung in 3 patients, kidney in 2 patients, melanoma in 2 patients and head and neck in 2 patients. Most of lesions (82%) were treated with the full prescription dose of 75Gy in 3 fractions, 8% with 67.5Gy (reduction dose of 10%), 5% of lesions with 60.0Gy (reduction dose of 20%) and 5% with 52.5Gy (reduction dose of 30%). At the time of SBRT, 21 (34.4%) patients presented with stable extrahepatic disease and 40 (65.6%) had no evidence of other sites of metastases.

All clinical cases were discussed by a liver multidisciplinary board including an expert hepatic surgeon. According to the international expert consensus [25] for all patients was defined the un-resectable status, considering the presence of one or more of following main factors.

### Extrahepatic disease

Twenty one (34.4%) patients presented un-resectable extra-hepatic disease at the time of SBRT

### Prior hepatic surgery

Twenty one (34.5%) patients were pre-treated with liver surgery and a second resection was not feasible

### Age and comorbidity

Twenty four (39%) are elderly patients and 40% of patients presented relevant comorbidities which did not allow the use of chemotherapy (16.4%) or the continuation beyond the first line of systemic therapies (24.6%)

### Lesions site

Thirty one (51%) patients presented central lesion, located close to the central biliary tract (HBC) and portal vein (PV).

According to the literature [26, 27] central lesions were defined as lesions located outside of the central liver zone, defined as a 2-cm expansion around the course of the portal vein contoured to its bifurcation in the liver. We treated 31 (51%) patients with a total of 40 (53%) central lesions with a prescription doses of 52.5Gy (4 lesions), 60Gy (6 lesions), 67.5Gy (60 lesions) and 75.0Gy (24 lesions).

The median FUP was 6.1 years (range 3–82 months).

**Fig. 1:**
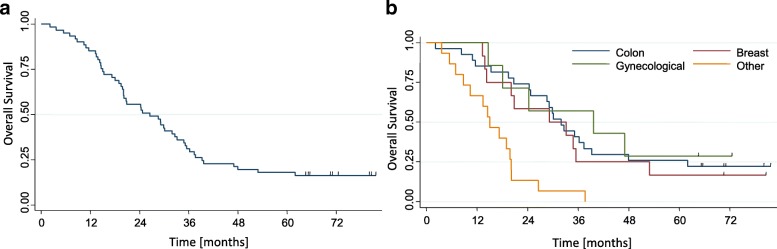
**a** Overall survival (OS); **b** OS by primary tumor

At the time of analysis, 10 patients (16%) are alive, without progression of disease. Most of the deceased patients (94%) died for progression of disease. The remaining patients died of non-cancer related causes. After SBRT, 12 (20%) patients presented intrahepatic progression only (of these 8 (13%) with extra-field progression and 4 (6%) with extra- and in-field progression), 17 (28%) patients showed extra-hepatic progression only and 16 (26%) patients presented both intra-hepatic and extra-hepatic progression of disease. Median OS was 27.6 months (95%CI: 23.2–34.8) and a range of 2.1–81.5 months and the survival rates were 85.2 ± 4.5%, 31.1 ± 5.9% and 18.0 ± 4.9% at 1, 3 and 5 year after SBRT, respectively. Figure 1a showed the Kaplan-Meier curve for OS. At univariate analysis, survival was significantly better in patients with favorable primary disease (colorectal, breast and gynecological cancer) compared to other primary sites of metastases (*p* = 0.001), as showed in Fig. 1b. The OS was independent of tumor size of liver metastases (*p* = 0.47) and the presence of stable extra-hepatic disease did not correlate to OS (*p* = 0.88). Patients with a single lesion presented with a 5 year OS of 28.6%, compared to 14.9% for patients with 2–3 lesion, however this difference was not significant (*p* = 0.22). Patients pre-treated with more than 2 line of chemotherapy (CHT) had a 5 year OS of 10.9% compared to 30.4% for those who received 0 or 1 line of CHT before the SBRT, but also this difference is not significant (*p* = 0.10). Table 3 summarized the univariate analysis results for OS.

**Table 3:** Univariate analysis for overall survival and local control

Covariate categories	N	OS (%)		*p*	LC (%)		*p*
		3 years	5 years		3 years	5 years	
Age, years							
> 70	24	25.0 ± 8.8	16.7 ± 7.6	0.57	64.2 ± 4.7	64.2 ± 5.9	0.71
< 70	37	35.1 ± 7.8	18.9 ± 6.4		82.3 ± 4.4	82.3 ± 5.6	
Gender							
Male	35	20.6 ± 6.9	11.8 ± 5.5	0.01	78.1 ± 4.3	78.1 ± 5.4	0.97
Female	26	44.4 ± 9.6	25.9 ± 8.4		81.8 ± 4.1	81.8 ± 5.4	
Cancer type							
Colorectal	29	41.1 ± 7.5	27.3 ± 8.1	< 0.001	75.3 ± 4.7	75.3 ± 5.7	0.47
Breast cancer	11	33.0 ± 7.3	20.1 ± 6.9		86.8 ± 4.8	86.8 ± 5.7	
Gynecological cancer	7	56.8 ± 6.9	27.8 ± 7.3		86.1 ± 4.7	86.1 ± 5.6	
Other	14	10.2 ± 7.2	–		80.0 ± 4.4	–	
Number of metastases							
1	48	42.8 ± 13.2	28.6 ± 12.1	0.22	82.0 ± 4.5	82.0 ± 5.4	0.28
2–3	13	27.7 ± 6.5	14.9 ± 5.2		67.3 ± 4.7	67.3 ± 5.6	
Size of metastases							
< 3 cm	32	28.1 ± 7.9	15.6 ± 6.4	0.46	77.2 ± 4.3	77.2 ± 5.5	0.60
> 3 cm	29	34.4 ± 8.8	20.7 ± 7.5		81.9 ± 4.3	81.9 ± 5.9	
Timing of metastasis							
Synchronous	24	29.3 ± 6.5	17.0 ± 5.5	0.93	–		–
Metachronous	37	32.2 ± 8.1	19.3 ± 6.9				
Time since diagnosis, mo							
≤ 12	35	31.4 ± 7.8	17.1 ± 6.4	0.91	–		–
> 12	26	30.8 ± 9.0	19.2 ± 7.7				
Prior local therapy							
Yes	27	33.3 ± 9.1	11.1 ± 6.1	0.54	65.8 ± 4.6	66.7 ± 5.7	0.06
No	34	29.4 ± 7.8	23.5 ± 7.3		87.1 ± 4.8	87.1 ± 5.8	
Pre-SBRT chemotherapy							
0–1 schedule	23	43.5 ± 10.3	30.4 ± 9.6	0.10	–		–
2–3-4 schedules	38	23.7 ± 6.9	10.5 ± 5.0				
Extrahepatic disease							
Yes	21	33.3 ± 10.3	14.3 ± 7.6	0.88	–		–
No	40	30.0 ± 7.2	20.0 ± 6.3				

**Fig. 2:**
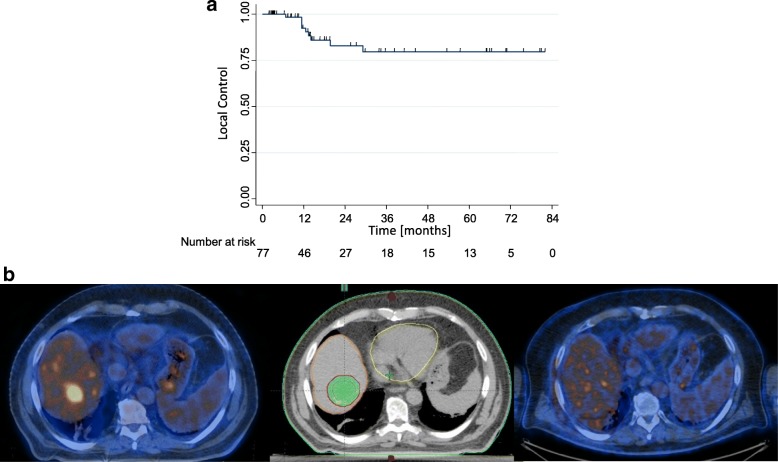
**a** Local control; **b** an example of a patient with complete response at 6 months from treatment

The LC rates at one, 3 or 5 were 94 ± 3.1%, 78.0 ± 5.9% and 78.0 ± 5.9%, respectively, the median LC time was not reached while the estimated mean LC time was 74.8 ± 3.9 months (95%CI: 67.1–82.5 months). Although cancer type was not related to local relapse, a sub group of breast and gynecological cancer patients had a very favorable LC rates of 86.1 ± 4.7% and 86.1 ± 5.6% at 5-year. For CRC liver metastases 5-year LC rate was 75%. Previous local ablative therapies were borderline significant for local control (*p* = 0.06). Liver metastases pre-treated with surgery or RFA presented with a 5-year LC of 65.8 ± 4.6, compared to hepatic lesions without prior local ablative treatments which had a LC rate at 5-year of 87.1 ± 5.8%. Patients with tumor size higher than 3 cm presented a local control at 5-year comparable to patients treated for smaller hepatic lesions (81.9% vs 77.2%; *p* < 0.60). Figure 2b showed a case of local control in 1 liver lesion treated with 75Gy in 3 fraction. Acute toxicity has been explained in a published preliminary report [16]. Analysis at 3 and 5-year confirmed the toxicity profile of our fractionation anticipated in the preliminary studies. As previously reported, only 1 patient experienced G3 late toxicity at 6 months, resulting from severe chest wall pain, which regressed within 1 year after treatment. In 2 patients G2 late toxicity was detected for appearance of moderate chest wall pain at 5 and 7 months, respectively. Concerning the healthy liver involvement, from the planning data, the mean liver dose was 12.7±5.1Gy and V15Gy was 1050±318cm^3^.

## Discussion

To date, the role of SBRT as ablative therapy of liver metastases is not consolidated and only recently, ESMO guidelines [28] added SBRT in the toolbox of local therapy for selected patients with CRC liver metastases unsuitable for surgery or RFA/MWA. All this is mainly related to the lack of long-term results from prospective and randomized trials. This report summarized the results of a phase II trial with more than 5-year follow-up for patients treated with high dose SBRT. The rational and the early results for the very high dose regimen proposed are summarized in our own earlier works [16, 17, 29].

Concerning treatment margins, the present study described the data from the original protocols which required relative large expansions from the CTV or ITV. With the current practice and with the knowledge that well-done abdominal compression, eventually associated to some additional breath control (e.g. breath hold) might allow to further reduce the margins and, consequently, to further protect the healthy fraction of the liver. This option was not part of the current investigation.

Table 4 summarizes the long-term outcome data of the few published SBRT trials with more than 40 enrolled patients and with a median follow-up longer than 2 years. The study by McPartlin [30], reported a 2 and 4-year LC rates of 49.8 and 26.2%, respectively for patients treated with 33 to 57Gy in 6 fractions. These results are worse than surgery and thermo-ablation and dose escalation is needed to improve sustained local control, as stated by the same authors. Similar conclusions were confirmed by Klement and by Joo, asserting that the use of ablative prescription dose is a crucial starting point to make SBRT a valid therapeutic option [31–33]. Joo [31] reported 2-year LC rates of 52, 83, and 89% using a biological equivalent dose (BED) ≤80Gy (group 1), 100-112Gy (group 2), and ≥ 132Gy (group 3), respectively. The authors concluded that better LC can be expected if higher doses are applied.

**Table 4:** Long term results of current studies on SBRT for liver metastases

Author, (reference) design study, year	Patients with liver metastases	Dose (Gy/ fr)	Follow-up (median)	Local control (%)		Overall survivall (%)		Toxicity ≥G3
				1-year	5-yeras	1-year	5-years	
Hoyer, [15] Phase II, 2006	44	45Gy/3fr	4.3 years	79% at 2 years	–	67%	13%	48%
Fode, [18] Retrospective. 2015	212 (321 tot oligometastatic patient)	BED10 = 72–195 Gy	5 years	91%	–	80%	23%	4%
				(Not specified for liver metastases)		(Not specified for liver metastases)		
Goodman, [39] Retrospective 2016	81	32–60/3–5 fr	2.25 years	96%	91% at 4 years	89.9%	28% at 4 years	4.9%
McPartlin, [28] Phase I-II, 2017	51	22.7–62.1 Gy /6 fr	2.3 years	49.8%	26% at 4 years	63%	9% at 4 years	3%
Joo, [29] Retrospective 2017	70	45-60Gy/3–4fr	2.8 years	93%	68% at 3 years	75% at 2 years		0%
Mendez Romero [40] Retrospective, 2017	40	37.5 Gy / 3fr	2.2 years	96%	66% at 3 years	95%	48% at 3 years	7.5%
		50.25 Gy / 3 fr		90%	81% at 3 years	94%	65% at 3 years	
Present study	61	75Gy/3 fr (82% of lesions) 67.5–52.5 Gy/3fr (18% of lesions)	6.1 years	94%	78%	85.2%	18%	1%

Klement [32, 33] suggested the use of doses greater than 3x17Gy and suggested to deliver a BED of 209 ± 67Gy to achieve a 2-year LC rate of 90% in patients with no prior chemotherapy and 286 ± 78Gy when chemotherapy was administered. The authors confirmed also the strong influence of histology and pre-treatment chemotherapy on LC, concluding that metastases from breast cancer respond better to SBRT compared to other histology types and that increased radiation resistance of CRC metastases may rather reflect the higher resistance after pre-SBRT chemotherapy. These innovative results could improve patient selection to liver SBRT and its efficacy, even though more prospective long-term data are needed.

The current prospective phase II study could therefore contribute to this search for evidence.

The fractionation employed in our trial escalates the dose with a BED ranging from 144.38Gy to 262.5Gy. Our results confirmed the safety and efficacy of ablative doses with a very high and sustained LC rate of 78% and with a very low toxicity profile after more than 5 years of follow up. Considering the suggestions of previously cited studies, our results showed also the effectiveness in disease progression control even in those unfavorable patients with bigger lesions (diameter > 3 cm), radio-resistant histology such as CRC and/or heavily pre-treated with systemic and local therapies.

The size of the lesions is a relevant factor affecting local control of ablative radiotherapy. Correlation between LC and lesion size higher than 3 cm was demonstrated by Rusthoven in a phase II trial on liver SBRT, using a dose prescription of 60Gy in 3 fractions [12]. Dose-response and size-response relationships were confirmed also in the recent report by McPartlin [30], with a 4-year local control rate of 26%, correlated with the GTV. Long-term results using the fractionation of 75Gy in 3 fractions showed that a promising LC can be also obtained in bigger lesions with a diameter ranging from 3 to 6 cm, with a 5-year LC rate of 81% as shown in the current study.

The high local response rate was not significantly influenced by any factors in our experience and CRC metastases presented a competitive LC rate at 5 years of 75%, although most of these patients were unfavorably selected. More than 50% of patients, indeed, received liver-directed therapy prior to SBRT and 86% of CRC patients were heavily pre-treated with systemic therapies before radiation treatment.

While the role of previous systemic treatments in inducing radio-resistance has been widely explained by Klement [32], the effect of previous local therapies is being studied.

In the last decades, the growth of cancer stem cells (CSC) in the site of previously local-treated tumors has been demonstrated. CSC seems to have the ability to self-renewal, to generate heterogeneous progeny and to self-divide unlimitedly. Considering these characteristics, CSCs seem to correlate to chemo- and radio-resistance development [35]. In our experience previous local ablative therapies were borderline significant for local control. This result could be explained by considering the limited number of enrolled patients in this trial and future studies on larger series could be needed to confirm this correlation.

Critical analysis of LC data confirmed the suggestion from Klement about a better response to SBRT of breast cancer liver metastases, with an optimal 5-year LC rate of 87%.

Primary tumor and previous therapies are crucial factors influencing the OS also. The efficacy of local ablative therapies as potentially curative for oligometastatic patients is consolidated for surgery of CRC liver disease [25, 34, 36–38], but it is debated for non-CRC liver metastases [﻿39﻿–﻿42﻿].

In a multicenter phase I/II study on patients treated with SBRT for liver metastases, Rusthoven [12] showed a 2-year OS of 30%. In this prospective experience, OS was better in patients with favorable cancer types (breast, CRC, sarcomas and renal cancer) than in patients with less favorable tumor types (lung, ovarian, and non-CRC gastrointestinal cancers), with a median OS of 32 months versus 12 months, respectively. These results promoted the efficacy of hepatic metastases ablation also for selected non-CRC liver metastases.

Although our study is not comparative and the primary endpoint is not OS, results show a benefit for the use of SBRT in the multidisciplinary management of oligometastatic patients with inoperable liver metastases not only from CRC, but also from other favorable primary disease, such as breast and gynecological cancers. In CRC liver metastases, indeed, our fractionation allowed an encouraging OS rate of 41% at 3 year and 27% at 5 year. Similar positive results were also achieved for patients with gynecological cancer-derived liver disease, with 3 and 5 year OS of 57 and 28%, respectively. Patients irradiated with breast cancer derived liver metastases showed a lower survival with a 3–5 year OS rates of 33–20%, respectively.

This apparently negative outcome could be related to an unfavorable selection of these patients. Indeed, patients with liver metastases from breast cancer, were pretreated with more systemic therapy before SBRT than those patients with other histological types. All 11 (100%) breast cancer patients received CHT and 8 (73%) patients were pretreated with more than 3 lines of systemic therapy. In this subgroup of patients, therefore, SBRT was more delayed.

These data confirmed that liver oligo-metastatic patients are often heavily pretreated and are unsuitable for the following systemic or local therapy at the time of referral for SBRT. As shown by Fode [19], the use of SBRT allows to decrease tumor progression in this setting of oligo-metastatic patients, with an encouraging 5-year OS rate of 23%, comparable to our result.

In our trial, all patients were unsuitable for surgery and RFA, 87% of patients received chemotherapy pre-SBRT and about 40% were heavily pre-treated with more than 3 lines of systemic therapy before SBRT. Supportive care remained the only therapeutic option for most of these patients, with an expected OS rate likely lower than 23% at 5-year [19].

Analyzing the current literature data on oligometastatic patients treated with ablative therapy, other prognostic factors for OS have been also identified beyond those of our study. A possible reason for this data could be given by patient selection.

All patients were enrolled in our trial according to restrictive inclusion criteria. They were defined by considering the importance of same prognostic factors suggested by surgical experiences. Since 1999, Fong showed the significant correlation between OS and specific variables, such as small number of lesions, size < 5 cm, absent or controlled extrahepatic disease, in patients with CRC liver metastases, treated with hepatic surgery [36]. Selection of patients at the time of enrollment, therefore, could justify why these variables do not improve OS in our experience. This remark seems to be confirmed by Chang in a pooled analysis on SBRT for CRC liver metastases [17]. In this experience, which employed inclusion criteria similar to our trial, number of lesions (1 vs 2–4), lesion size and active non-liver disease were not correlated with OS, as well.

The need to identify simple predictors of outcome, an important task of state-of-the-art research, was investigated by Mazzola [43]. The authors proposed the use of diagnostic signatures from PET imaging as predictors of local response in liver metastases. These included pre-SBRT SUV-max and SUV-mean. Considering the relatively low sample size, our long term results suggest that patients selected by favorable primary disease, number, size and site of liver metastases and controlled extrahepatic disease, could benefit from high dose of SBRT also in terms of OS.

The data presented in our report refer to linac based SBRT. The use of alternative delivery modalities, like, e.g., robotic devices as CyberKnife has been investigated. A review from Ihnat [44] showed that the most frequently treated are for less than 5 metastases with a maximum of 6 cm and no extrahepatic disease. In general, severe toxicity is rare and local control range from 70 to 100% at 1 year and from 60 to 90% at 2 years.

As a final remark, it should be advised that, thy type of very high-dose stereotactic regime should be carried out in appropriately qualitied and experienced centers and that sufficient quality assurance procedure are enforced to guarantee the safety of the treatments.

## Conclusions

The 5-year results confirmed the role of high-dose SBRT in the treatment of liver metastases with diameter > 3 cm, which are often unsuitable for other effective local ablative therapy. Prognostic factors identified suggested the relevant role of cases selection and the timing of SBRT in the therapeutic pathway of these patients.

## Abbreviations

4D-CT: Four dimensional computed tomography
BED: Biological equivalent dose
CHT: Chemotherapy
CI: Confidence interval
CRC: Colorectal cancer
CSC: Cancer stem cells
CT: Computed tomography
CTV: Clinical target volume
EORTC: European Organization for Research and Treatment of Cancer
ESMO: European society for medical oncology
GTV: Gross tumor volume
IGRT: Image guided radiation therapy
ITV: Internal target volume
LC: Local control
MRI: Magnetic resonance imaging
MU: Monitor unit
MV: Mega voltage
MWA: Microwave ablation
OAR: Organ at risk
OS: Overall survival
PET: Positron emission tomography
PFS: Progression free survival
PTV: Planning target volume
RECIST: Response Evaluation Criteria In Solid Tumors
RFA: Radiofrequency ablation
RILD: Radiation induced liver disease
SBRT: Stereotactic body radiation therapy
SUV: Standardized uptake value
